# Diffraction limited photonic hook via scattering and diffraction of dual-dielectric structures

**DOI:** 10.1038/s41598-021-99744-5

**Published:** 2021-10-13

**Authors:** Victor Pacheco-Peña, Joseph Arnold Riley, Cheng-Yang Liu, Oleg V. Minin, Igor V. Minin

**Affiliations:** 1 School of Mathematics, Statistics and Physics, Newcastle University, Newcastle Upon Tyne, NE1 7RU UK; 2 School of Engineering, Newcastle University, Newcastle Upon Tyne, NE1 7RU UK; 3 Department of Biomedical Engineering, National Yang Ming Chiao Tung University, Taipei, 112 Taiwan; 4 National Research Tomsk Polytechnic University, 30 Lenin Avenue, Tomsk, Russia 634050

**Keywords:** Applied optics, Terahertz optics

## Abstract

Photonic hooks have demonstrated to be great candidates for multiple applications ranging from sensing up to optical trapping. In this work, we propose a mechanism to produce such bent structured light beams by exploiting the diffraction and scattering generated by a pair of dielectric rectangles immersed in free space. It is shown how the photonic hooks are generated away from the output surface of the dielectrics by correctly engineering each individual dielectric structure to generate minimum diffraction and maximum scattering along the propagation axis. Different scenarios are studied such as dual-dielectric structures having different lateral dimensions and refractive index as well as cases when both dielectrics have the same lateral dimensions. The results are evaluated both numerically and theoretically demonstrating an excellent agreement between them. These results may open new avenues for optical trapping, focusing and sensing devices via compact and simple dual-dielectric structures.

## Introduction

An exciting prospect of structured light^1,2^ is to *bend the rules* of physics by tailoring the entire field but considering only a region of interest within it^3^. One such example is the recent discovering of the so-called photonic hooks^4,5^. In this context, it has been shown that light passing through mesoscaled (sizes comparable to the incident wavelength) dielectric objects with broken symmetry can produce a near-field sub-wavelength scale localized curved beam. Interestingly, such beams have minimal beamwaist smaller than half of the wavelength in the surrounding medium, yet they only require wavelength-sized asymmetric structures^3^. The asymmetry of such structures can be related to different factors such as asymmetry in terms of the external shape of the particle, an asymmetry of the refractive index while using a symmetric particle width, or a completely symmetric particle (both in refractive index and shape) with an asymmetry of the illuminating light^6–9^. A detailed comprehensive review of works in this area is analysed in^3^.

From a historical point of view, the presence of two foci produced by spherical and cylindrical particles with dimensions comparable to the size of the wavelength was investigated in^10–12^. These studies gained the attention of the scientific community and gave rise to the now known “photonic jets”^13–19^. One of the first detailed studies of the localization of optical radiation by a rectangular phase step was carried out in^20^. It was shown that by engineering the height and width of a phase step (with a refractive index of 1.46) to be approximately equal to the wavelength of the incident radiation (355 nm), it is possible to generate a localized spatial region of increased intensity, i.e., a photonic jet. Moreover, in^21^ it was shown that the diffraction of a wave on a rectangular step forms a curvilinear hyperbolic localized region of radiation (i.e., a curved photonic jet). Recently it has been shown that the focal length of a dielectric structure based on a pair of dielectric rectangular bars with equal refractive index depends on the separation distance between the dielectric particles^22^. By combining the refractive index contrast, separation distance, and dimensions of the dielectric bars, it was shown that diffraction-limited focusing can be achieved.

Inspired by these recent findings along with the interesting opportunities that photonic hooks can offer in different applications such as sensors and optical trapping^3^, in this work we propose and demonstrate the possibility of generating structured light beams such as photonic hooks by using a pair of dielectric rectangular particles with different dimensions and/or refractive index. An attractive feature of such a structure is its extreme simplicity, compactness (using rectangular shapes), and the possibility of dynamically controlling the photonic hook parameters, for example, by measuring the distance between the structures.

## Results

### Focusing electromagnetic waves with geometrically asymmetric dielectrics

**Figure 1:**
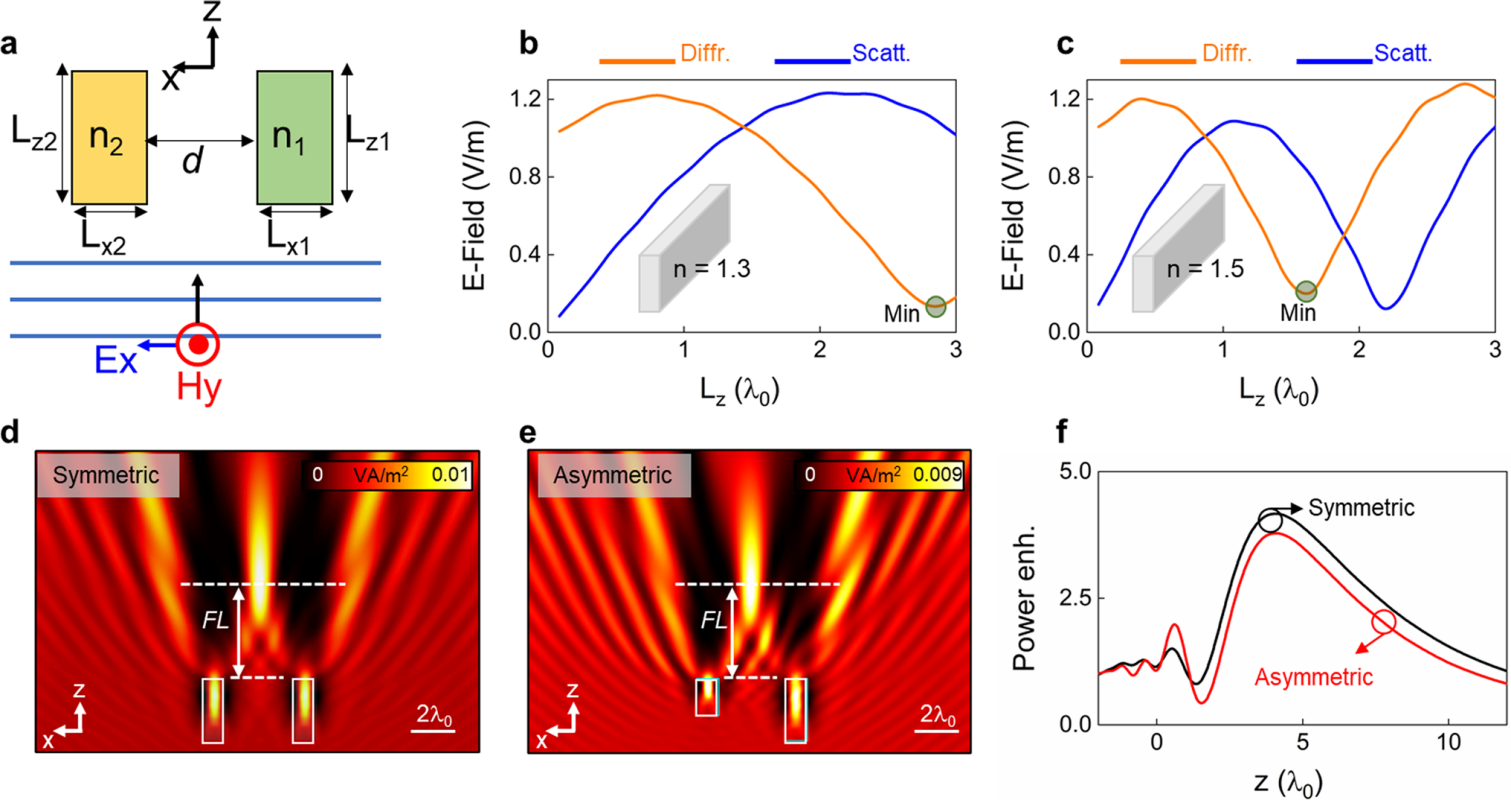
(**a**) Schematic representation of the proposed dual-dielectric configuration to generate a photonic hook when illuminated with a planewave. Note that the position *z* = 0 is located at the output surface of the two dielectrics (see *x-* and *z-* axes in this panel). (**b**, **c**) numerical results of the scattered (blue lines) and diffracted (orange lines) field distributions generated by a single dielectric particle immersed in free-space calculated at a distance *z* = 4*λ*_*0*_ from the output surface of the dielectric with lateral dimension *L*_*x*_ = *λ*_*0*_ and variable *L*_*z*_. In these results we consider dielectric particles having a refractive index of *n* = 1.3 (**b**) and *n* = 1.5 (**c**). (**d**) Power distribution on the *xz* plane of a dual-dielectric structure with the same dimensions and refractive index (i.e., symmetric case with n_1_ = n_2_ = 1.3 and *L*_*z1*_ = *L*_*z2*_ = 2.83*λ*_*0*_, these values correspond to the minimum scattered field as shown in panel (**b**)). (**e**) Power distribution on the *xz* plane of a dual-dielectric structure with different dimensions and refractive index (i.e., asymmetric configuration with n_1_ = 1.3 and *L*_*z1*_ = 2.83*λ*_*0*_ and n_2_ = 1.5 and *L*_*z2*_ = 1.67*λ*_*0*_, again these values correspond to the minimum scattered field as shown in panels (**b**, **c**) respectively). (**f**) Power enhancement along the propagation *z-*axis calculated at *x* = *y* = 0 for the symmetric (black) and asymmetric (red) dual-dielectric configurations shown in (**d**, **e**) respectively. Panel (**a**) was created using Microsoft PowerPoint version 2102.

To begin with, the schematic representation of the proposed dual-dielectric structure for the generation of photonic hooks is shown in Fig. 1a. We consider two dielectrics with refractive index *n*_*1,2*_ separated by a distance *d* and being immersed in free space (*n*_*0*_ = 1). Without loss of generality we consider 2-dimensional (2D) rectangular shapes for the dielectrics having dimensions *L*_*x1,2*_ and *L*_*z1,2*_ along the *x-* and *z-*axis, respectively. With this setup, the dual-dielectric structure is excited with a TM planewave under normal incidence (*E*_*x*_) which propagates along the *z-*axis. From the point of view of structure miniaturization, in this work, we will consider 2D dielectric rectangular bars with dimensions comparable to the wavelength of the incident wave (i.e., mesoscaled structures).

Before studying the creation of photonic hooks with the proposed dual-dielectric structure, let us first analyze the scattering and diffraction features of a single dielectric particle^22^ immersed in free space, being illuminated with a planewave. To do this, we fix the width of the dielectric to be *L*_*x*_ = *λ*_*0*_ at the operational wavelength of *λ*_*0*_ = 3 mm (0.1 THz) and vary the dimension along the propagation direction (*L*_*z*_) from 0 to 3*λ*_*0*_. The scattering and diffraction produced by such a single dielectric particle are numerically evaluated using the transient solver of the commercial software CST Studio Suite® where top and bottom magnetic boundary conditions are implemented to consider a 2D dielectric particle^14,23^. The magnitude of the scattered field is calculated as |***E***_***scatt***_| =|***E***_***background—***_***E***_***total***_| with ***E***_***background***_ and ***E***_***total***_ as the numerically calculated electric field distribution without and with the presence of the dielectric, respectively^24^. Finally, the diffracted field |***E***_***diffr***_| was obtained by simply recording the magnitude of the electric field along the propagation *z-*axis using ***E***_***total***_. With this setup, the numerical results of the scattered and diffracted field distribution as a function of *L*_*z*_ (ranging from 0 to 3*λ*_*0*_) for a single dielectric immersed in free space are shown as blue and orange curves, respectively, in Fig. 1b, c considering a refractive index of the dielectric of *n* = 1.3 (Fig. 1b) and *n* = 1.5 (Fig. 1c). These results are calculated at a distance *z* = 4*λ*_*0*_ away from the output surface of the dielectric particle. As it can be observed, different peaks and dips of scattering/diffraction are obtained depending on the refractive index of the dielectric, as expected.

As reported in Ref.^22^, such scattering/diffraction properties of single dielectric particles can be exploited to focus an incident planewave into a focal spot by using a pair of dielectrics having the same refractive index and geometrical dimensions. For completeness, we provide an example of such focusing structure in Fig. 1d by considering two dielectric particles with a refractive index of *n* = 1.3 and dimensions *L*_*x1,2*_ = *λ*_*0*_ and *L*_*z1,2*_ = 2.83*λ*_*0*_ separated by a distance *d* = 3*λ*_*0*_ (i.e., a total distance of 4*λ*_*0*_ measured from the center of both dielectric particles). Note that the value of *L*_*z1,2*_ = 2.83*λ*_*0*_ is selected such that it corresponds to the length of the dielectric along the propagation *z-*axis that produces the smallest diffraction at the position *z* = 4*λ*_*0*_. Now, an interesting question can be asked: would it be possible to generate a similar focusing device as in Fig. 1d but considering two dielectric particles with different refractive index and dimensions along the propagation *z-*axis (i.e., *n*_*1*_* ≠ n*_*2*_ and *L*_*z1*_ ≠ *L*_*z2*_, respectively)? To answer this question, we can simply consider the single dielectrics from Fig. 1b, c and select their dimensions *L*_*z1*,2_, such that both of them generate a minimum diffracted electric field at the same position as the design from Fig. 1d (*z* = 4*λ*_*0*_). With this in mind, we use *n*_*1*_ = 1.3 with *L*_*z1*_ = 2.83*λ*_*0*_ and *n*_*2*_ = 1.5 with *L*_*z2*_ = 1.67*λ*_*0*_ for the right and left dielectric particles, respectively. The numerical results of the power distribution on the *xz* plane of this dual-dielectric structure are shown in Fig. 1e where it can be observed how a similar focus to that of a symmetric configuration (Fig. 1d) is obtained. To better compare these results, we provide in Fig. 1f the numerical results of the power enhancement (calculated as the ratio of the power with and without using the two dielectrics) along the propagation *z-*axis for both scenarios presented in Fig. 1d, e. As observed, both symmetric and asymmetric structures can generate a focus at exactly the same position (focal length *FL* ~ 4.08 *λ*_*0*_) with a power enhancement of 4.1 and 3.8, respectively, demonstrating how it is indeed possible to emulate the focusing structure from Fig. 1d by carefully designing an asymmetric dual-dielectric structure with the same diffraction properties of each individual dielectric.

### Full asymmetric dual-dielectric structures

Based on the result from Fig. 1 one can ask, could we further exploit the asymmetric configuration discussed in Fig. 1e to generate a photonic hook? As discussed in Fig. 1d, e, a focus can be produced by considering two dielectric particles with dimensions and refractive index chosen such that each of them produces minimal diffraction along the propagation *z*-axis. However, as a photonic hook is a bent beam, we can exploit full asymmetric dielectric structures by properly selecting the dimensions (*L*_*z1,2*_) and refractive index (*n*_*1,2*_) of the right and left dielectric particles, respectively, such that one of them can produce a maximum scattering while the other particle generates minimum diffraction along the *z*-axis.

**Figure 2:**
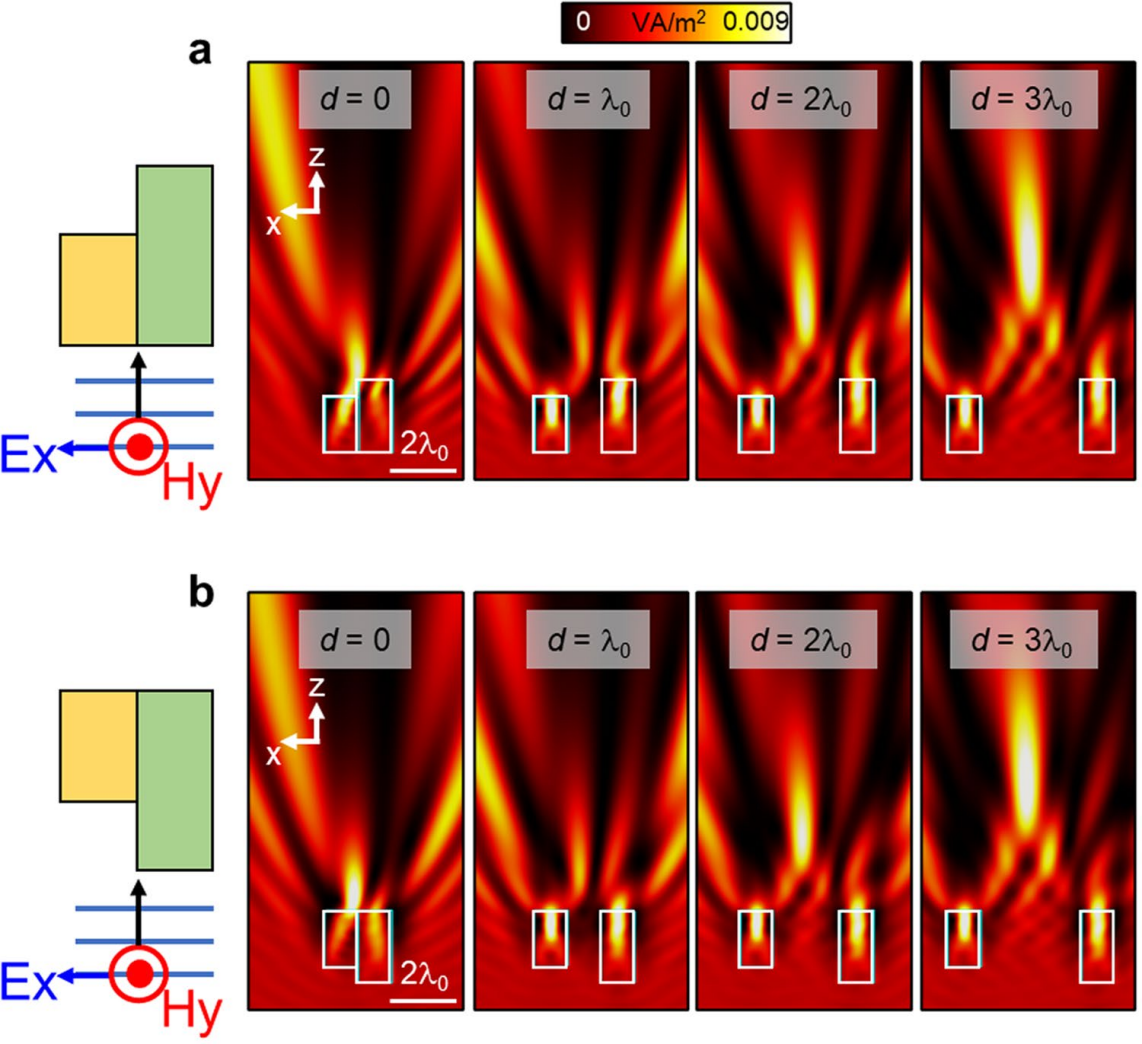
Examples of photonic hooks using full asymmetric dual dielectric structures. (**a**, **b**) Numerical results of the power distribution on the *xz* plane for a dual-dielectric structure designed to produce maximum scattering (right dielectric) and minimum diffraction (left dielectric) at *z* = 4*λ*_*0*_ from the output surface of each individual dielectric. The dielectrics have a refractive index of n_1_ = 1.3 and n_2_ = 1.5 with a length along the propagation *z-*axis calculated from Fig. 1b, c of *L*_*z1*_ = 2.17*λ*_*0*_ and *L*_*z2*_ = 1.67*λ*_*0*_, respectively. We consider different values of *d* ranging from 0 up to 3*λ*_*0*_ and two configurations: (**a**) dielectrics aligned at their input surface and (**b**) dielectrics aligned at their output surface.

We provide different examples of such full asymmetric dual-dielectrics structure in Fig. 2. Note that the configuration shown in Fig. 2a with rectangular particles of different dielectric materials and equal size without a separation between the two particles was analysed in^8^. Here we use the same dielectrics as in Fig. 1b, c with *n*_*1*_ = 1.3 and *n*_*2*_ = 1.5 for the right and left dielectrics, respectively. The dimension along *z* for each dielectric is extracted from Fig. 1b, c, respectively, by considering maximum scattering (*L*_*z1*_ = 2.17*λ*_*0*_) and minimum diffraction (*L*_*z2*_ = 1.67*λ*_*0*_) at *z* = 4*λ*_*0*_. With this configuration, the numerical results of the power distribution on the *xz* plane using different distances between the dielectrics (from 0 to 3*λ*_*0*_ with a step of *λ*_*0*_) are shown in Fig. 2a, b considering that the dielectric particles are aligned at their output or input surface, respectively. From these results, one can notice how a photonic hook is obtained in all the cases. However, for small distances (*d* = 0) the photonic hook mainly depends on the refracted wave produced by the dual-dielectric structure. When the parameter *d* is increased, a clearer bent light beam is achieved which is due to the interaction of the diffraction/scattering patterns from both particles. As expected, shown in Fig. 2a, b, a clear photonic hook is observed when *d* = 3*λ*_*0*_ in both scenarios.

**Figure 3:**
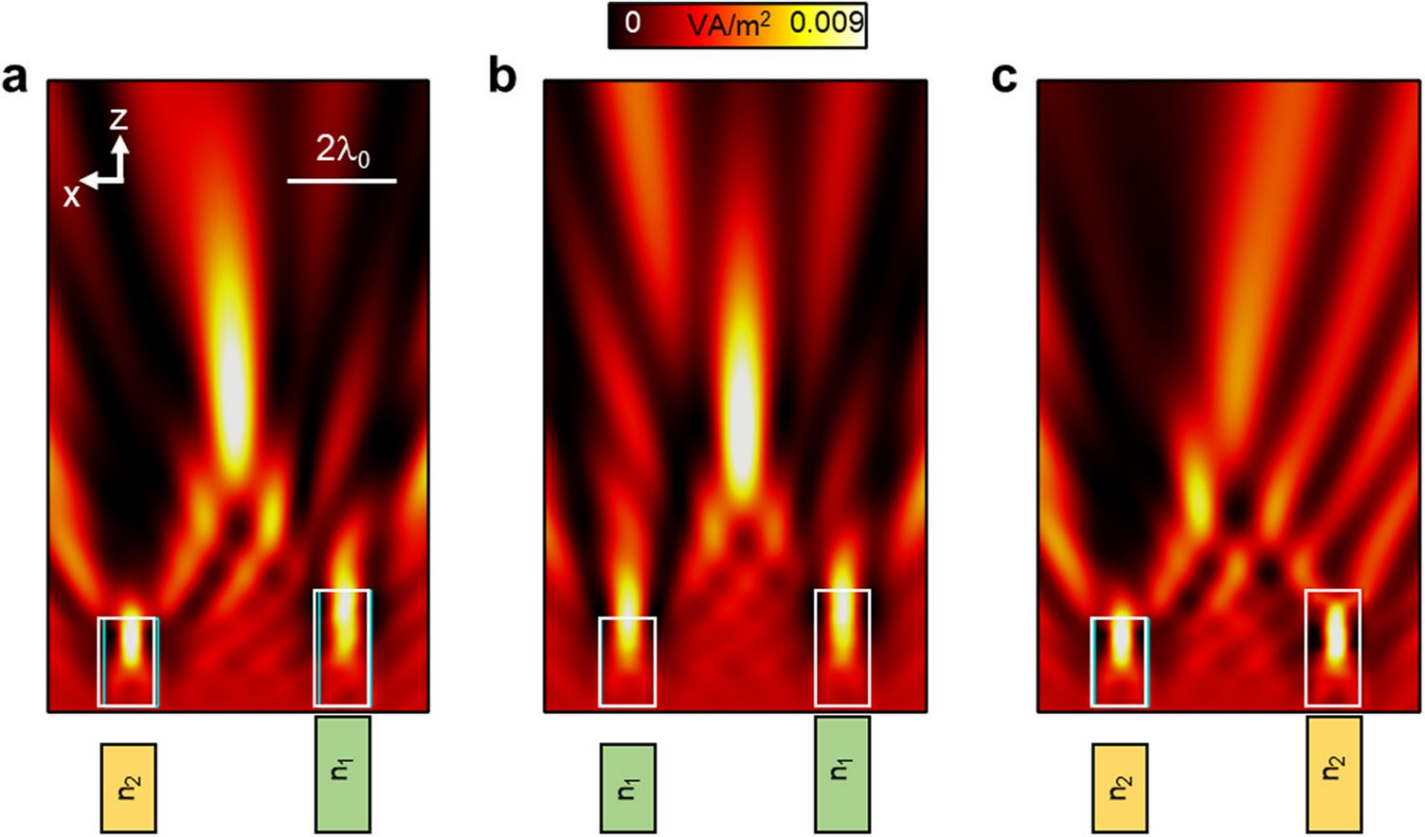
Numerical results of the power distribution on the *xz* plane for the dual-dielectric structure shown in Fig. 2a considering: (**a**) same results as those shown in the last panel of Fig. 2a with *d* = 3λ_0_ where both dielectrics have a different refractive index, (**b**) when the dielectrics have a refractive index of n_1_ = n_2_ = 1.3 and when they are (**c**) n_1_ = n_2_ = 1.5.

Finally, it is important to highlight that, as the full asymmetric dual-dielectric structure shown in Fig. 2 was designed by exploiting maximum and minimum scattering and diffraction for each particle, respectively, its response will depend on both the geometry and refractive index of the dielectrics, as expected. To better observe this, we can consider the case shown in Fig. 2a with *d* = 3*λ*_*0*_ and change the refractive index of the particles to be either equal to n_1_ or n_2_. The results for each case are shown in Fig. 3b, c, respectively. Note that we have again included the results for the full asymmetric dual-dielectric structure in Fig. 3a for the sake of completeness. As observed, a photonic hook is not achieved in the configurations shown in the middle and right panels of Fig. 3, demonstrating the importance of both geometry and refractive index when using a full asymmetric dual-dielectric structure.

### Single asymmetric dual-dielectric structures

Now, in the studies shown in Figs. 2, 3 we have discussed how it is possible to generate a bent light beam by using a pair of fully asymmetric dielectric particles (i.e., both with different geometries and refractive index). Based on this one may ask, would it be possible to produce photonic hooks by exploiting the scattering and diffraction produced by a pair of dielectric particles with the same dimensions but different refractive index, i.e., a dual-dielectric structure with a single asymmetry as shown in Fig. 1a? To answer this question, we can follow a similar approach as in the previous studies where the scattering and diffraction of each single dielectric particle are first studied.

**Figure 4:**
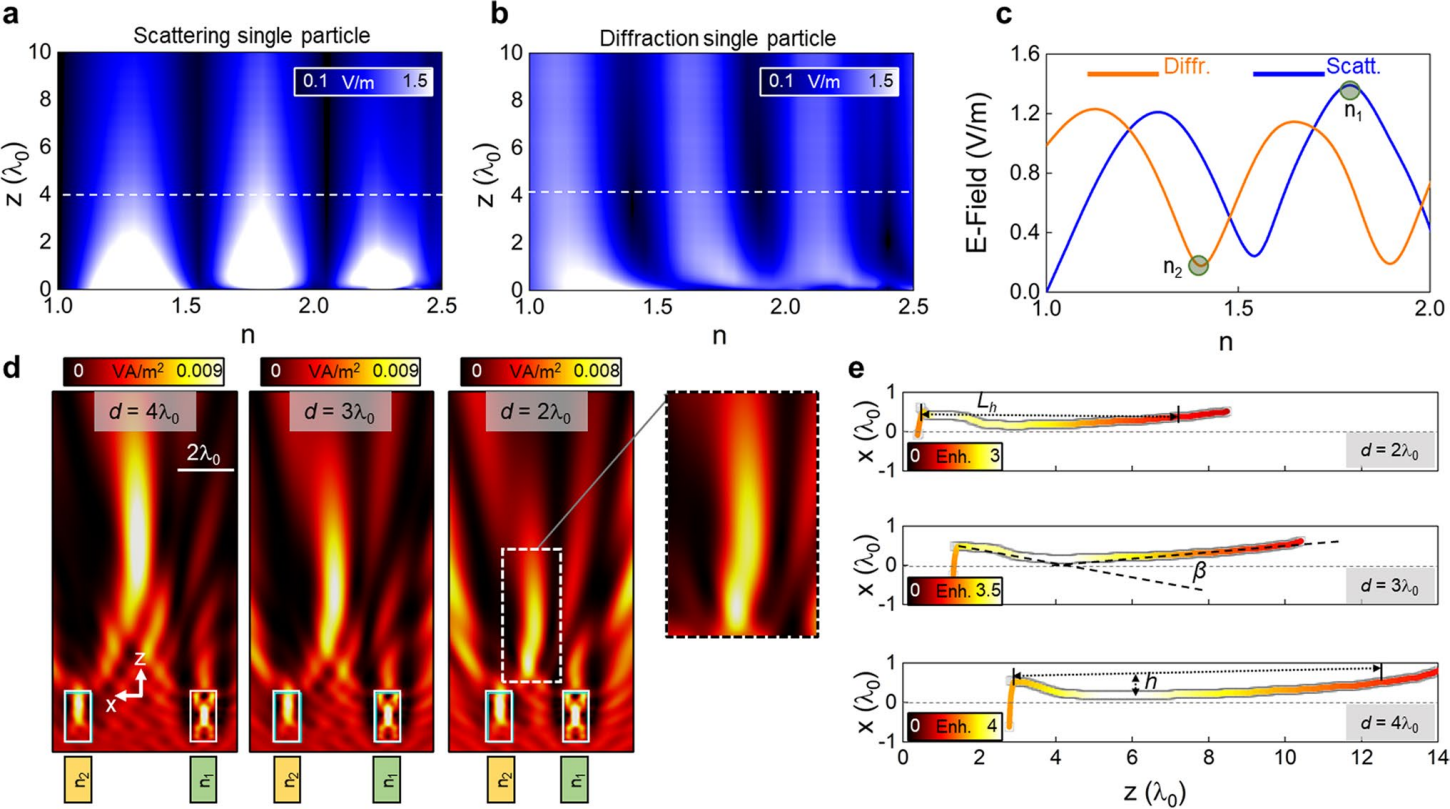
(**a**, **b**) Numerical results of the scattered and diffracted field distributions, respectively, along the propagation *z*-axis generated by a single dielectric particle with lateral dimension *L*_*x*_ = *λ*_*0*_ and *L*_*z*_ = 2*λ*_*0*_ with variable refractive index, *n*, immersed in free-space. (**c**) Scattered (blue line) and diffracted (orange line) extracted from the dashed white lines of panels a and b, respectively, at *z* = 4*λ*_*0*_. (**d**) Numerical results of the power distribution on the *xz* plane of a dual-dielectric structure with both dielectric particles having the same lateral dimensions *L*_*x1,2*_ = *λ*_*0*_ and *L*_*z1,2*_ = 2*λ*_*0*_ and refractive index values of n_1_ = 1.8 and n_2_ = 1.4 to generate maximum scattering and minimum diffraction, respectively (see panel **c**). The distance *d* between the dielectrics is *d* = 4*λ*_*0*_ (left), *d* = 3*λ*_*0*_ (middle) and *d* = 2*λ*_*0*_ (right). The inset in this latter panel corresponds to a zoom-in-image of the field distribution for the case with *d* = 2*λ*_*0*_. (**e**) Numerical results of the (*x, z*) positions of the maximum power enhancement for the dual-dielectric structures with *d* = 4*λ*_*0*_ (bottom), *d* = 3*λ*_*0*_ (middle) and *d* = 2*λ*_*0*_ (top).

Here we will consider again a single 2D dielectric rectangle immersed in air and dimensions *L*_*x*_ = *λ*_*0*_ and *L*_*z*_ = 2*λ*_*0*_ along the *x-* and *z*-axes, respectively. In this study, we will focus on the effects of a variation of the refractive index of the dielectric particle instead of its length along the *z-*axis as we aim to design a dual-dielectric structure with both dielectrics having the same spatial dimensions, as shown in Fig. 1a. With this setup, the scattering, *|E*_***scatt***_*|*, and diffracted, *|E*_***diffr***_*|*, field distribution are calculated along the propagation *z*-axis (from *z* = 0 to *z* = 10*λ*_*0*_) at *x* = *y* = 0 and the numerical results are shown in Fig. 4a, b, respectively. As observed, both the scattering and diffraction present peaks and dips (as in Fig. 1b, c) depending on the refractive index of the 2D rectangle. Interestingly, the position of these peaks and dips do not change for positions *z* > 1.5*λ*_*0*_. These results are of particular importance in our case as the aim of the proposed device from Fig. 1a is to produce a bending light beam away from the output surface of the dual-dielectric structure (*z* = 0). For completeness, we provide in Fig. 4c the numerical results of the scattering and diffracted field distributions at *z* = 4*λ*_*0*_ extracted from the white dashed lines in Fig. 4a, b, respectively.

As discussed in Figs. 2, 3, we can produce a photonic hook by designing a dual-dielectric structure such that one of the dielectric particles produces a high scattering while the other minimum diffraction. With this in mind, and following Fig. 4a–c, we can fix the dimensions of both dielectrics to be equal (*L*_*x1*_ = *L*_*x2*_ = *λ*_*0*_ and *L*_*z1*_ = *L*_*z2*_ = 2*λ*_*0*_ as shown in Fig. 1a) but select their refractive index as *n*_*1*_ = 1.8 (right dielectric) and *n*_*2*_ = 1.4 (left dielectric) to engineer the required maximum scattering and minimum diffraction, respectively (see Fig. 4c). With this configuration, the numerical results of the power distribution on the *xz* plane for the dual-dielectric structure with equal dimensions but different refractive index are shown in Fig. 4d considering different distances between the dielectrics (namely *d* = 4*λ*_*0*_, *d* = 3*λ*_*0*_ and *d* = 2*λ*_*0*_). From these results, a clear photonic hook is obtained away from the output surface of the dielectrics, as expected. To better compare these results, we extracted the position of the maximum power enhancement on the *xz* plane and the results are shown in Fig. 4e along with the power enhancement represented as the colored lines. From these results, the (*z, x*) coordinates of the numerical *FL* for the structures with *d* = 4*λ*_*0*_, *d* = 3*λ*_*0*_ and *d* = 2*λ*_*0*_ are (5.75*λ*_*0*_, 0.19*λ*_*0*_), (3.9*λ*_*0*_, 0.15*λ*_*0*_) and (2.5*λ*_*0*_, 0.18*λ*_*0*_), respectively, with a power enhancement at the focus of ~ 4, ~ 3.6 and ~ 2.7 for the same values of *d*, respectively. Note that, similar to the results discussed in Fig. 1, the position of the focus along the propagation axis for the photonic hooks in Fig. 4d, e clearly depends on the distance between the dielectrics as the total distance between particles (measured from the center of both particles) is 5*λ*_*0*_, 4*λ*_*0*_, and 3*λ*_*0*_ for the cases shown in Fig. 4d, respectively, which are close to the values of the *FL*, as mentioned above. Moreover, from the results shown in Fig. 4e, one can observe how the photonic hook is elongated when increasing the distance *d* between the dielectrics, in agreement with the results shown in Fig. 2, with a larger power enhancement for larger values of *d*, as detailed above. In this context, the length of the photonic hook (defined by the subtense *L*_*h*_ and calculated as the distance between the *FL* and the points on the *xz* plane at which the power enhancement has decayed 1/*e* value^25^, Fig. 4e) is *L*_*h*_ ~ 12.4*λ*_*0*_, *L*_*h*_ ~ 10.3*λ*_*0*_ and *L*_*h*_ ~ 7*λ*_*0*_ for the cases with *d* = 4*λ*_*0*_, *d* = 3*λ*_*0*_ and *d* = 2*λ*_*0*_, respectively, demonstrating how the photonic hook is more elongated for larger values of *d*, as suggested above.

Regarding the curvature of the bent light beams^25^, we calculate the curvature parameter *β* as in^26^ (see schematic representation on Fig. 4e) resulting in values of *β* ~ 14°, *β* ~ 15° and *β* ~ 17° when separating the dielectric particles by a distance *d* = 4*λ*_*0*_, *d* = 3*λ*_*0*_ and *d* = 2*λ*_*0*_, respectively. Finally, we also extracted the height of the photonic hook by calculating the parameter *h* (defined as the distance between the *FL* and the subtense *L*_*h*_^25^, see schematic representation in Fig. 4b) resulting in values of *h* ~ 0.55 *λ*_*0*_, *h* ~ 0.35 *λ*_*0*_*,* and *h* ~ 0.13*λ*_*0*_ again for *d* = 4*λ*_*0*_, *d* = 3*λ*_*0*_ and *d* = 2*λ*_*0*_, respectively. These results corroborate that the photonic hook becomes more elongated when increasing *d* while its curvature is slightly reduced compared with smaller distances *d* between the two dielectrics. Note that here the height *h* is reduced when considering smaller distances *d* between the dielectric particles. This is due to the fact that (i) the photonic hook decays faster along the propagation direction for smaller distances *d* and (ii) the curvature of the photonic hook at the *FL* is less pronounced compared to cases when the curved beam is generated near the surface of a dielectric (as reported in^25^). For completeness, the spatial resolution of the photonic hooks is calculated by obtaining the Full-Width at Half-Maximum (FWHM_x_, defined as the distance along the transversal *x*-axis at which the power enhancement has decayed half its maximum) at the position of the maximum power enhancement along the propagation *z-*axis from Fig. 4d, e. The resulting values are FWHM_x_ = 0.76*λ*_*0*_, FWHM_x_ = 1.23*λ*_*0*_ and FWHM_x_ = 1.37*λ*_*0*_ for the same distances between the dielectrics (*d* = 2*λ*_*0*_, *d* = 3*λ*_*0*_ and *d* = 4*λ*_*0*_, respectively), demonstrating how diffraction-limited photonic hooks can be designed with the proposed dual-dielectric structures.

**Figure 5:**
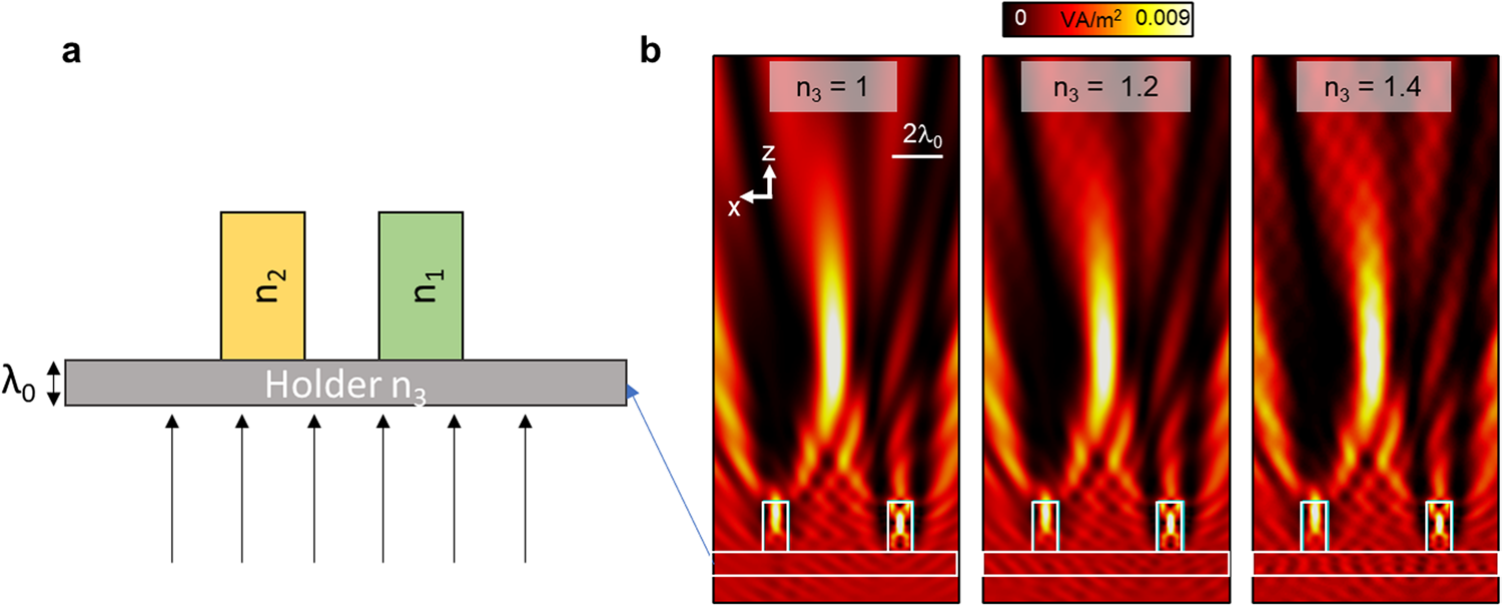
(**a**) Schematic representation of the proposed dual-dielectric structure on top of a dielectric holder of refractive index n_3_ as a potential experimental structure. (**b**) Numerical results of the power distribution on the *xz* plane for the design discussed in Fig. 4d with *d* = 4*λ*_*0*_ when using a dielectric holder with a thickness of *t* = *λ*_*0*_ and refractive index n_3_ = 1 (left), n_3_ = 1.2 (middle) and n_3_ = 1.4 (right), respectively. Panel (**a**) was created using Microsoft PowerPoint version 2102.

Finally, the results discussed in Figs. 1, 2, 3, 4 have been carried out considering the two dielectric particles to be free-standing and immersed in air. However, to ease the validation of such scenarios in potential experimental demonstrations, it is interesting to evaluate the response of the proposed structures by placing them on top of a dielectric plane acting as a holder (see Fig. 5a for a schematic representation of the potential experimental structure). With this setup, the numerical results of the power distribution on the *xz* plane considering different materials for the dielectric holder are shown in Fig. 5b with n_3_ = 1, n_3_ = 1.2 and n_3_ = 1.4 (from left to right, in the same figure respectively). Here we consider the same dimensions parameters of the dual-dielectric structure as those shown in Fig. 4d with *d* = 4*λ*_*0*_. As observed, a photonic hook is clearly observed in all the cases, demonstrating how such a configuration could be potentially used in future experimental demonstrating.

## Conclusions

In conclusion, we have proposed and demonstrated dual-dielectric structures with the ability to generate diffraction-limited photonic hooks at distances away from their output surface when illuminated with a planewave. It has been shown how such bent light beams can be produced by correctly engineering the dimensions and refractive index of the two dielectrics such that one of them is able to generate a large scattering while the other a low diffraction along the propagation *z*-axis. Different configurations have been studied both numerically and theoretically such as dual and single asymmetric dielectric particles demonstrating a good agreement between them. For completeness, we have also proposed a potential experimental structure consisting of a pair of dielectric rectangles placed on top of a dielectric holder, demonstrating that photonic hooks are also expected to be excited with this configuration. The results shown here have been carried out considering low THz frequencies (0.1 THz) but can be directly extended to any frequency range from acoustics, microwaves up to the optical regime.

## Methods

The numerical results were carried out using the transient solver of the commercial software CST Studio Suite®. The 2D dielectric particles (of height 0.2*λ*_*0*_) were immersed in a vacuum box of dimensions 27*λ*_*0*_ × 0.2*λ*_*0*_ × 27*λ*_*0*_ along the *x-, y-* and *z*-axis. Top and bottom magnetic boundary conditions were used (to consider the whole simulation space as a 2D) and open-add-space was used at the front, back, left, and right boundaries of the simulation box. The dielectric particles were illuminated with a TM planewave (*E*_*x*_). Finally, a refined hexahedral mesh was implemented with a minimum and maximum mesh size of 0.02*λ*_*0*_ and 0.0373*λ*_*0*_, respectively. The results of the scattered field from Fig. 1b, c were calculated by implementing an electric field monitor at the design wavelength (*λ*_*0*_). Then, two simulations were performed: one without and one with the dielectric rectangles. This allowed us to calculate the background (*E*_*background*_) and total (*E*_*total*_) electric field distributions, respectively. Finally, the scattered field was calculated as |*E*_*background—*_*E*_*total*_|. The diffracted field |*E*_*diffr*_| from Fig. 1b, c was obtained by recording the magnitude of the electric field along the propagation *z-*axis using *E*_*total*_.
